# The Treatment of Tubercular Enteritis

**Published:** 1893-09-09

**Authors:** G. A. Sutherland

**Affiliations:** Physician to the North London Consumption Hospital


### THE TREATMENT OF TUBERCULAR ENTERITIS (*concluded*).

By G. A. Sutherland, M.D.Ed., M.R.C.P.Lond., Physician to the North London Consumption Hospital.

II. *Medicinal.*—While the most lasting benefit is undoubtedly due to judicious dieting, the progress of the patient may also be greatly helped by means of medicines. Of these the most valuable is the subnitrate of bismuth, given as a powder. To gain the full effect the amount given must be large, one and a half to two drachms, or even more, in the twenty-four hours. An ordinary dose is twenty grains every four hours, and, if it is practicable, a more frequent administration, such as ten grains every two hours, seems to act better. If there is sickness present, a dose administered an hour before a meal will often prevent vomiting. The effect of the bismuth is to soothe the inflamed mucous membrane, and also by its antiseptic action to check fermentation and putrefaction. Amongst astringent medicines for checking the diarrhœa the most valuable are coto and hæmatoxylin, and if there is much abdominal pain present opium may be added. The following prescription is found serviceable: ℞ tr. coto, ♏xv.; tr. opii, ♏x.; tr. aurantii, ♏xx.; dec. hæmatoxyli, ad. ℥j. Sig.: One ounce thrice daily after food. When the motions are very offensive $\beta$-naphthol is prescribed as follows: ℞ $\beta$-napthol, ʒj.; pulv. tragac co., ʒj.; tr. aurantii, ʒvj.; aquam, ad. ℥vj. Sig.: One tablespoonful thrice daily after food. It is not absorbed, and as it passes through the intestine acts as a local antiseptic. In the early stages tonic medicines are better avoided, with the exception of maltine, which is usually well tolerated, and at the same time aids digestion. One or two teaspoonfuls may be given three times a day after food.

III. *Local.*—In some cases in which sickness is persistent it is found advantageous, before commencing the dietetic treatment, to wash out the stomach with warm water. Abdominal pain or discomfort is often present, and this is relieved by the application of hot flannel fomentations containing half an ounce of turpentine, or the abdomen may be covered with a mixture of equal parts of belladonna extract and glycerine. It is also extremely important to have the rectum thoroughly cleared out by an enema once or twice a week. The presence of scybalous masses in the rectum will reflexly excite peristalsis in the intestine, and thus the diarrhœa is maintained. It is useful to add carbonate of soda to the fluid used in the injection (half an ounce to one pint), so as to soften and remove the excessive secretion of mucus present in the rectum. As the subjects of tubercular enteritis are extremely susceptible to cold, a flannel belt ought to be worn round the abdomen.

IV. *Hygienic.*—The first stage of the treatment is best conducted in hospital, where the patient can be kept in bed and the diet administered as directed until the diarrhœa has ceased and the patient is able to retain and digest his food. He is then allowed out of bed, and if the weather is favourable he is encouraged to be out of doors as much as possible, with the caution that he must not over-exert himself. The stage now reached, as far as the local disease is concerned, is that the intestinal catarrh has been checked and fermentation has ceased, but the ulceration still remains, and is beyond the reach of direct medication. Further progress in the way of cicatrisation must depend on the prevention of digestive troubles, and the improvement of the general health by constitutional treatment.

A change to the country or seaside is most beneficial, and at the same time the patient must be carefully instructed as to his diet, so as to prevent relapses, which are by no means uncommon. Tonics may be given more freely, such as codliver oil, quinine and iron, nux vomica, &c. The diet must be as nourishing as possible, and often milk, which proves so disastrous during the stage of acute ulceration, can be taken in large quantities with marked benefit. Like all forms of tubercular disease, this affection is chronic in character, and great care must be taken for some months after all signs of active mischief have ceased.

